# Antineoplastic effect of piperine compared with doxorubicin via suppressing γ-secretase/notch pathway in breast cancer cells

**DOI:** 10.1007/s12672-026-05165-z

**Published:** 2026-07-12

**Authors:** Maha M. Salem, Hamed A. Abosharaf, Marian N. Gerges, Mohamed A. Abd El-Moneim, Tarek M. Mohamed, Aliaa M. Radwan

**Affiliations:** 1 Biochemistry Division- Chemistry Department, Faculty of Science, Tanta University, Tanta, 31257 Egypt; 2 Biochemistry Department, Faculty of Dentistry, Sinai University, Arish Branch, North Sinai, 45511 Egypt

**Keywords:** Breast cancer, Piperine, Doxorubicin, Notch pathway, γ-secretase

## Abstract

Breast carcinoma is the most prevalent and lethal malignancy affecting females, and it always presents an essential healthcare problem. Doxorubicin (DOX) is a common chemotherapy utilized in treating breast carcinoma, but it also has dangerous side effects. Hence, searching for potent and safe anticancer compounds is urgent. An alkaloid, piperine, mostly found in pepper species, is a naturally occurring substance with anticancer properties. This work aims to assess the anticancer impact of piperine on breast carcinoma cells by potentially inhibiting the γ-secretase/Notch pathway, and to compare its impacts with those of DOX. The anti-proliferative effects of piperine and DOX were evaluated on various human breast carcinoma cell lines (MCF-7, T47D, and MDA-MB-231). Additionally, a flow cytometric analysis was carried out to identify the cell cycle arrest. Moreover, the protein expression levels of Notch-1, γ-secretase components, NICD, c-myc, p21, Bax, and Bcl-2 were assessed. The results demonstrated that among the tested cell lines, MDA-MB-231 cells exhibited the highest sensitivity to piperine treatment, with the lowest IC_50_ value (32.37 ± 2.5 µM). At the G2/M phase, piperine and DOX both stopped the cell cycle. Furthermore, piperine and DOX inhibited the Notch signaling by reducing Notch-1, γ-secretase components, and NICD expression. Piperine and DOX also reduced the levels of Notch target proteins, namely, p21 and c-myc. Lastly, both drugs induced apoptosis by elevating Bax and reducing Bcl-2 expression in malignant breast cells. The promise of piperine as a targeted therapy candidate for the treatment of breast cancer is substantially supported by this research.

**Graphical Abstract:**

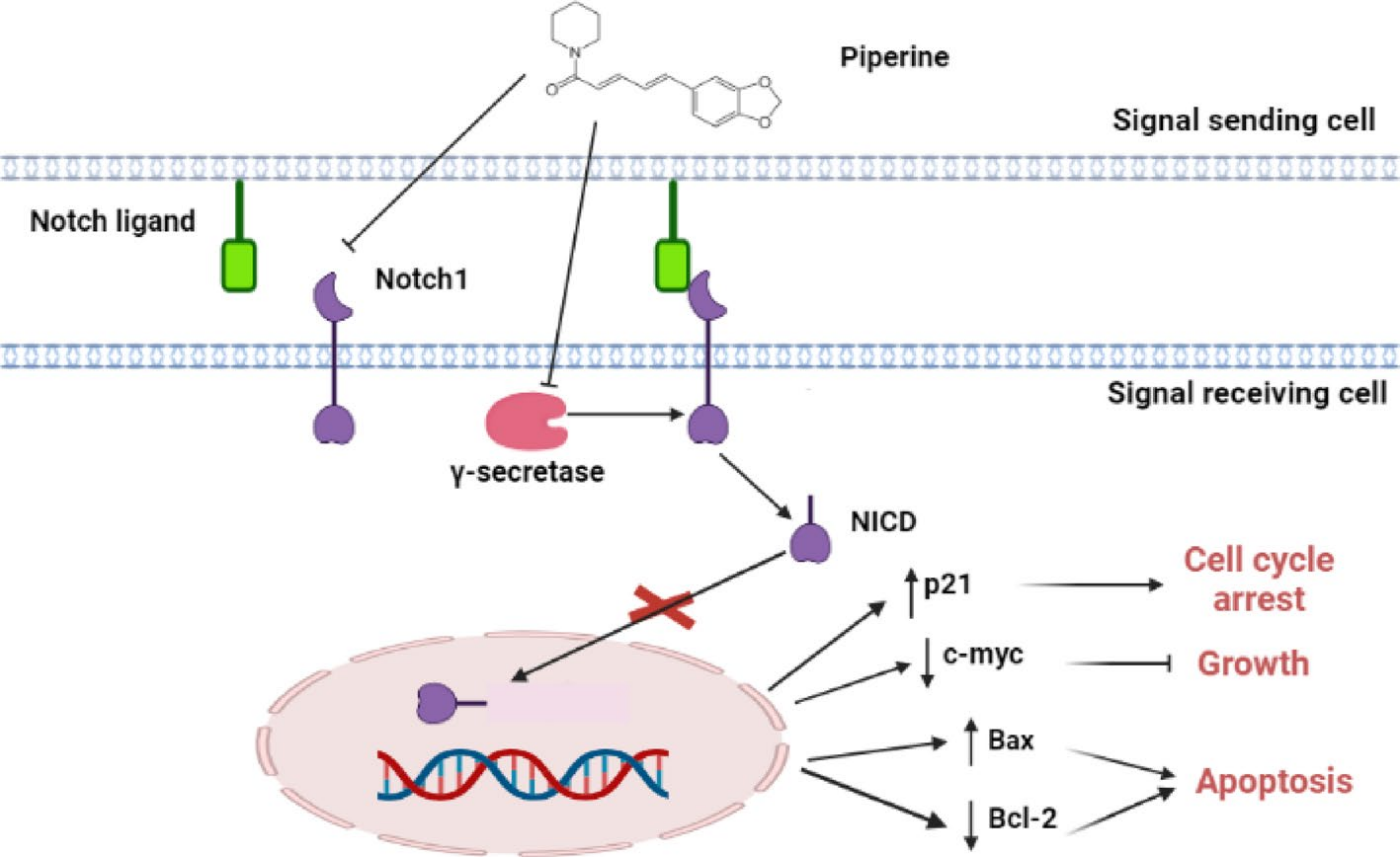

**Supplementary Information:**

The online version contains supplementary material available at 10.1007/s12672-026-05165-z.

## Introduction

Breast cancer stands as one of the most widespread malignancies and is a major cause of mortality among women. The most recent data available indicates that 670,000 women worldwide died because of breast cancer in 2022, while 2.3 million received a diagnosis [1]. By 2040, there will be more than 3 million new cases of breast cancer identified each year. This increase is particularly apparent in developing countries, where access to early diagnosis and treatment may be less efficient than in developed countries [2]. Understanding the complex nature of breast carcinoma is essential to creating efficient medications [3]. To increase treatment effectiveness and decrease side effects, research is still being done on targeted therapies that focus on particular genetic abnormalities or molecular markers linked to breast cancer [4].

Abnormal activation of the Notch signaling has been linked to breast cancer development, progression, invasiveness, metastasis, and resistance to treatment [5]. Notch signaling, to put it briefly, is a mechanism of communication between cells that involves a Notch receptor (Notch1, 2, 3, or 4) and its ligands, either Delta-like ligands (DLL1, DLL3, and DLL4) or Jagged 1–2 (JAG1 and JAG2) [6]. Furin protease, ADAM family proteases, and the γ-secretase complex are the three main proteases that activate the Notch signaling pathway. Furin protease, situated upstream of the Notch pathway, aids in the Notch receptor maturation. The Notch receptors are cleaved by the ADAM family proteases, thereby releasing the Notch extracellular domain (NECD). The Notch receptors’ intracellular domains are finally cleaved by the γ-secretase, a multiprotein complex. After γ-secretase cleavage, the Notch intracellular domain (NICD) is liberated, reaches the nucleus, starting the transcription of downstream genes [7]. γ-secretase inhibitors act by keeping γ-secretase closed, making it incapable of cleaving substrates like Notch and other proteins [8].

Numerous disadvantages of the chemotherapy medications currently in use include metastasis, recurrence, non-selectivity to cancer cells, and multidrug resistance [9]. The anthracycline antibiotic, doxorubicin (DOX), was first used clinically in the 1970 s as part of chemotherapy treatments for breast carcinoma and other malignancies. Despite being an excellent anticancer medication, DOX has serious dose-limiting side effects, including cardiotoxicity. DOX is characterized by numerous cytotoxic processes, including oxidative stress and free radicals generation, topoisomerase II poisoning, DNA intercalation and adduct formation, and membrane destruction via altered sphingolipid metabolism [10]. Crucially, DOX often causes chemoresistance in patients with breast carcinoma, especially those with triple-negative breast cancer [11]. Therefore, the development of safe medications is urgently needed.

Piperine, the chief pungent alkaloid present in black and long peppers, exhibits a range of pharmacological effects, including cardiovascular protection, neuroprotective, antioxidant, anti-fungal, anti-bacterial, and anti-inflammatory properties [12]. Additionally, piperine exhibited anticancer activities against breast, cervical, prostate, lung, skin, stomach, liver, colorectal, and bone cancers [13]. Numerous signaling pathways linked to breast cancer therapy are targeted by piperine, including cell-cycle arrest, apoptosis induction, modulation of signaling protein expression, and downregulation of transcription factors. It can also enhance the bioavailability of chemotherapeutic medications [14].

Although many studies have investigated the capability of various natural products in modulating the Notch signaling pathway in breast cancer cells, the effect of piperine on inhibiting the Notch pathway, particularly γ-secretase expression, has not yet been studied. Hence, this research aims to investigate the antiproliferative impact of piperine against various breast cancer cell lines. This investigation will focus on piperine’s inhibitory role in the γ-secretase/Notch signaling pathway. DOX, a widely used chemotherapeutic agent in breast cancer management, was included as a reference drug to benchmark the anticancer effects of piperine against an established clinically FDA-approved therapy.

## Materials and methods

### Chemicals and drugs

Piperine (CAS Number: 94-62-2, purity: 98%, sourced from Acros Organics, New Jersey, USA) was used in this study. Doxorubicin HCl was obtained as Doxilyd 50 mg from Celon Laboratories Ltd., India. All other chemicals were of high quality.

### Molecular docking and ADMET profiling

Molecular docking was performed to predict the potential interaction and binding of both piperine and the reference medication DOX with the target protein γ-secretase. After downloading the crystal structure of the target protein (PDB: #6IYC) (https://www.rcsb.org/structure/6IYC) from the Global Protein Data Bank, the structure was refined and optimized. This procedure entailed energy minimization using the (MM2) force field after co-crystallized ligands and undesirable water molecules were eliminated [15]. Using Chem-Draw Ultra 8.0 (https://en.freedownloadmanager.org/users-choice/Chemdraw_Ultra_8.0.html), the two-dimensional (2D) structures were created. Subsequently, these structures were converted into three-dimensional (3D) motif files. Ligands and the target protein interactions were analyzed utilizing Molegro Virtual Docker (http://molexus.io/molegro-virtual-docker/). The Piecewise Linear Potential (PLP) algorithm is the scoring function in computational screening. The MolDock simplex evolution search algorithm operates with a grid resolution of 0.30 Å. Among the ten poses generated, they were arranged based on their MolDock Scores, and for further analysis, the pose with the lowest score was chosen. Additionally, the bioavailability and drug-likeness of the compounds were assessed using the online ADMETlab 2.0 tool (https://admetmesh.scbdd.com/) [16].

### Cell lines and culture conditions

The human breast cancer cell lines MCF-7 (#ATCC HTB-22), T47D (#ATCC HTB-133), and MDA-MB-231 (#ATCC HTB-26) were obtained from the American Type Culture Collection (ATCC) company via the CERMA Center, Alexandria University, Egypt, and were used throughout the study. These cell lines were cultured in DMEM medium supplemented with 10% FBS and 1% penicillin/streptomycin in a 37 °C incubator with 5% CO_2_ [17–19].

### Cell treatment and viability assay

A stock solution of piperine was prepared in 0.1% dimethyl sulfoxide (DMSO) and consequently added to a fresh medium to achieve the desired final concentrations for cell treatment. Cell viability was assessed using the MTT assay [20]. Briefly, in 96-well plates, cells have been seeded at a density of 1 × 10^4^ cells/well. These cells were then exposed to varying doses of piperine and DOX. After a 48-hour incubation, MTT (5 mg/mL in PBS) has been added to each well, followed by an additional 4-hour incubation at 37 °C in a cell culture incubator. Subsequently, the supernatant was removed, and 100 µL of DMSO was added to each well; then the plate was shaken for 15 min. The absorbance was determined at 630 nm by a microplate reader (Bio-Rad, CA, USA).

### Cell cycle analysis

Flow cytometry-based cell cycle analysis was conducted to examine the impact of piperine and DOX on MDA-MB-231 cell cycle progress, following a previously described method [21]. After being cultivated at a density of 1 × 10^5^ cells in six-well plates, the cells were incubated for a full day. Following a 48-hour treatment with the IC_50_ of piperine and DOX, cells were collected, washed with 1x PBS, and preserved in 70% ice-cold ethanol throughout the whole night at −20 °C. After centrifugation, cells were resuspended in PBS containing 10 mg/ml RNase A. Following a 30-minute incubation at 37 °C, cells were treated with a 50 µg/mL solution of propidium iodide (PI) and kept in the dark for 30 min at room temperature. The DNA content of labeled cells was determined by means of Cell Quest Software with an Accuri C6 flow cytometer (Becton Dickinson, Franklin Lakes, BD, USA). PI fluorescence was detected for data gathering and analysis using the FL2 channel (585/40 nm bandpass filter) utilizing a linear scale, essential for precise DNA content resolution. The gating technique was implemented hierarchically as outlined below. An initial scatter gate was established on a forward scatter (FSC) vs. side scatter (SSC) dot plot to identify the intact cell population while excluding cell debris (events with minimal FSC/SSC values) and big aggregates (events with maximal FSC/SSC values). Secondly, doublet discrimination was executed by plotting FL2-Area (FL2-A) against FL2-Width (FL2-W) to solely gate single cells and exclude cell doublets, which may otherwise artificially elevate the G2/M and S-phase fractions due to their comparable DNA content to two diploid cells. Third, within the singlet gate, a PI fluorescence histogram was produced on a linear scale, allowing for the identification and quantification of distinct cell cycle populations.

### Western blotting assessment

Immunoblotting investigations were conducted as previously described [22]. The ReadyPrepTM protein extraction kit (163–2086) from Bio-Rad Inc was used for protein isolation in accordance with the manufacturer’s instructions. The total protein concentration was quantified using the Bradford Protein Assay Kit (SK3041) from Bio Basic Inc. (Markham, Ontario L3R 8T4 Canada). Subsequently, equivalent amounts of protein (20 µg) were separated on 12% sodium dodecyl sulfate (SDS)–polyacrylamide gels and electroblotted onto polyvinylidene fluoride (PVDF) membranes. The membranes were blocked with 5% skimmed milk for 1 h at room temperature, and then primary antibodies with dilutions (1:1000) were added. Primary antibodies used were against presenilin1 (PSEN1, A00138-1), presenilin enhancer (PEN2, GTX85063), p21 (ab227443), Notch1 (E-AB-12815), Nicastrin (NBP2-19538), NICD (Cleaved Notch1, #4147), c-myc (E-AB-30975), Bcl-2 (sc-7382), Bax (sc-7480), APH-1 (A04859). The membranes were incubated overnight at 4 °C. Afterward, the blots were detected with horseradish–peroxidase-conjugated anti-rabbit IgG (1:5000) dilution using a chemiluminescent substrate (Clarity TM Western ECL substrate Bio-Rad, cat#170–5060) and visualized using a charge-coupled device (CCD) camera-based imager. To ensure equivalent loading, membranes were subsequently reprobed with β-actin antibodies. Band intensities were quantified by normalizing each band’s density to that of β-actin.

### Statistical analysis

The data are accessible as mean ± SE. To compare treated and untreated cells, one-way ANOVA followed by Tukey’s test in GraphPad Prism Software 6 (San Diego, CA, USA) was performed. Probability values below 0.05 were reflected statistically significantly.

## Results

### Molecular docking of piperine and DOX with the γ-secretase target notch protein and ADMET features

**Fig. 1:**
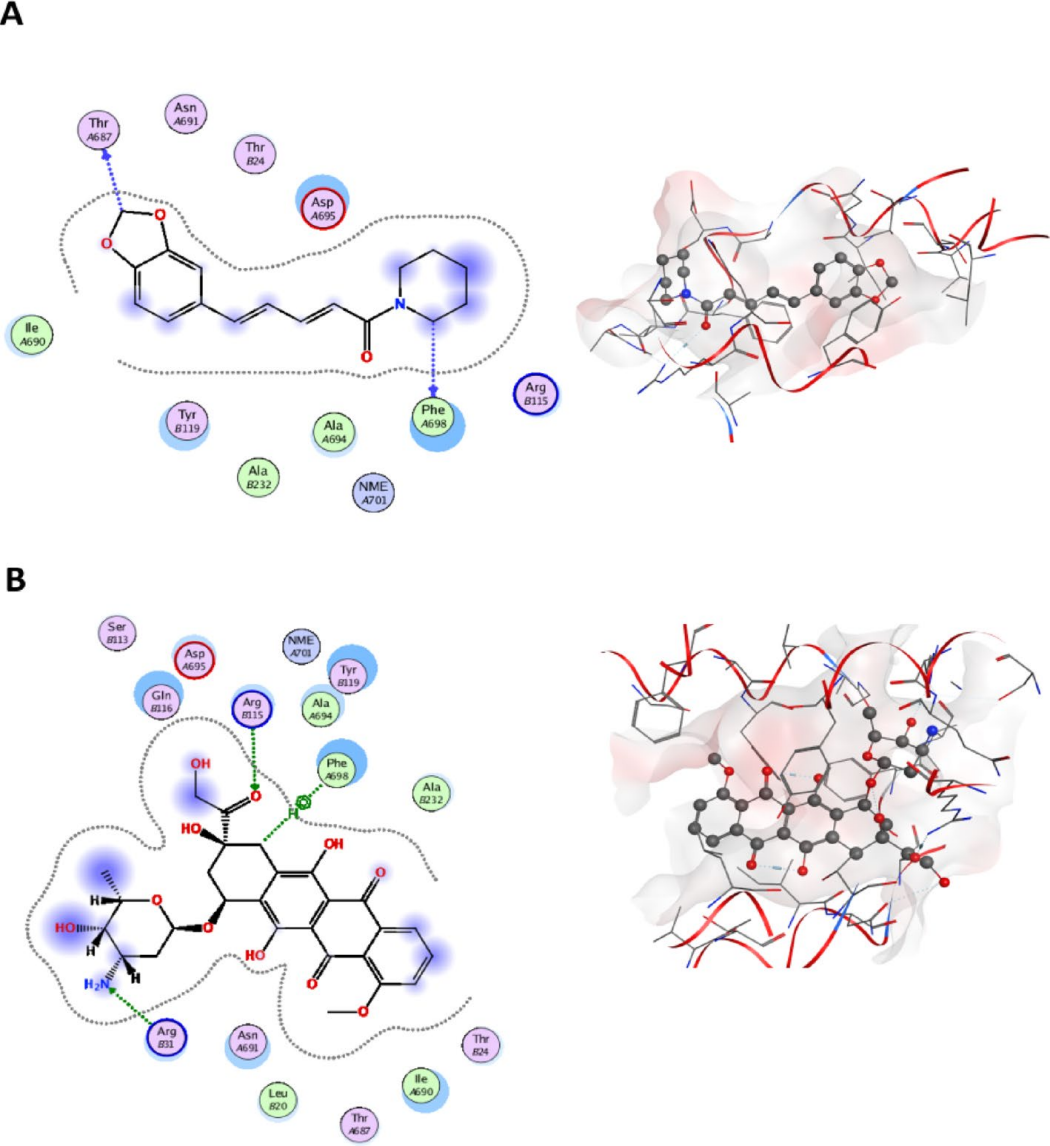
The molecular docking simulations of **A** piperine and **B** doxorubicin (DOX) towards the γ-secretase target protein

To gain theoretical insights into molecular interactions, piperine and DOX were docked against the γ-secretase complex, a key enzyme in the Notch signaling pathway. The docking scores of piperine and DOX against the γ-secretase protein were − 6.6284 and − 8.2541 kcal/mol, respectively, which predicted favorable binding affinities for both compounds. The docking simulations suggested that piperine interacts with the γ-secretase primarily via a hydrogen bond (acceptor) with the amino acid residue ARG B115. Additionally, several potential electrostatic interactions were identified with residues including ALA B232, TYR B119, GLN B116, ALA A700, PHE A698, ASP A695, ILE A690, ALA A694, LEU B20, ASN A691, THR B24, and THR A687. In comparison, DOX exhibited a higher predicted affinity, potentially mediated by hydrogen bonds (acceptor) with ARG B31 and ARG B115, alongside a non-covalent interaction with PHE A698. Further electrostatic interactions were identified with ASP A695, GLN B116, ASN A691, ILE A690, ALA B232, ALA B694, and TYR B119 **(**Table 1 and Fig. 1).

**Table 1:** The molecular interactions of piperine and doxorubicin (DOX) against the **γ-**secretase target Notch protein

Compounds	Docking score (ΔG_bind_)	Docked complex (amino acid–ligand) interactions		Distance (Å)
		Involved receptor residues	Type of interaction bond	
Piperine	−6.4823	THR A687 PHE A698	H-donor	3.08 2.63
		ASP A695 ILE A690 TYR B119 ALA A694 ARG B115	Electrostatic interactions	
DOX	−8.2541	ARG B31 ARG B115	H-acceptor	3.1 3.31
		PHE A698	H-pi	4.11
		ASP A695 GLN B116 ASN A691 ILE A690 ALA B232 ALA B694 TYR B119	Electrostatic interactions	

**Fig. 2:**
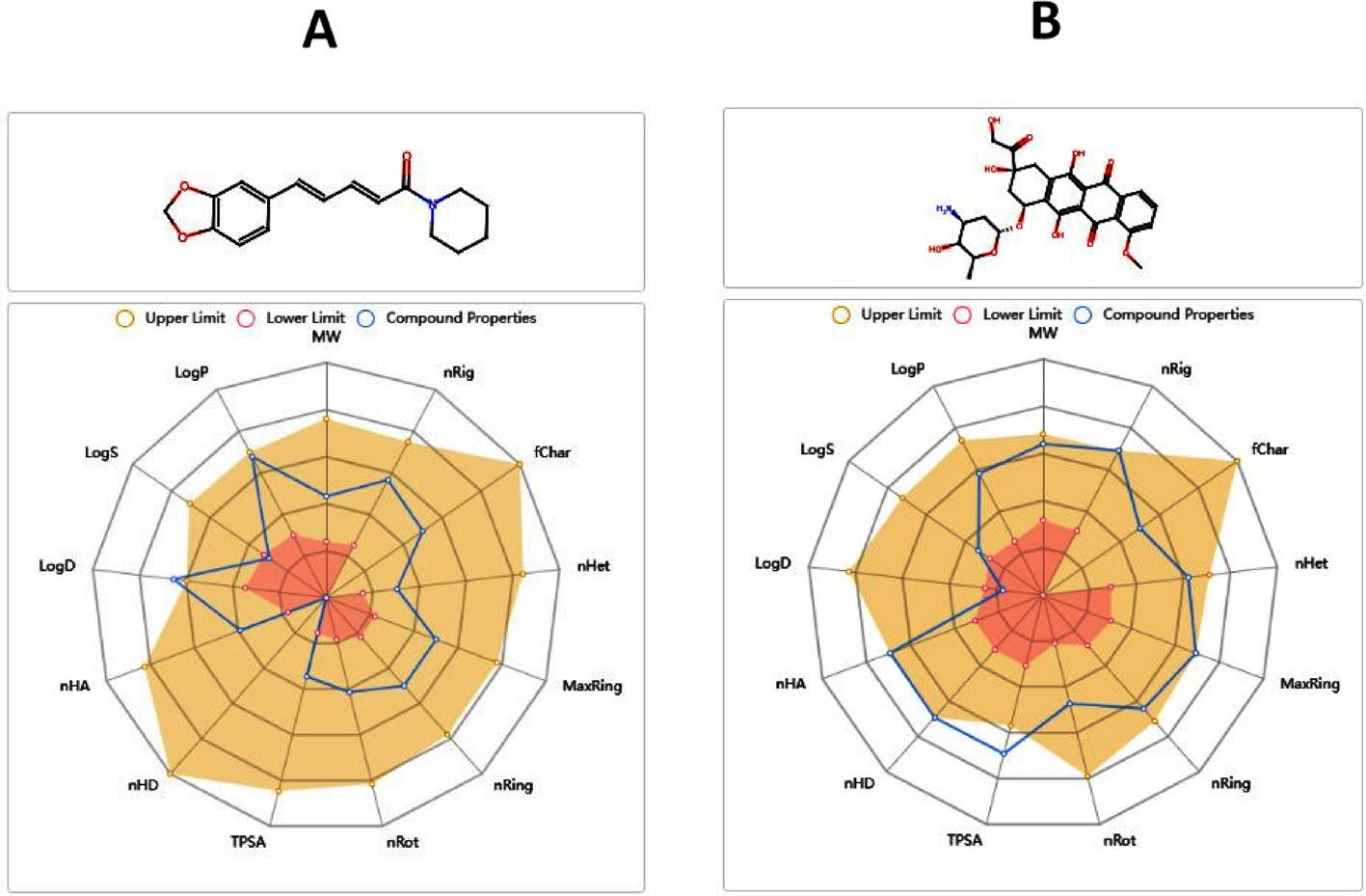
ADMET features of **A** piperine and **B** doxorubicin (DOX)

Regarding ADMET features, the results showed that only piperine follows Lipinski’s rule. Additionally, topological polar surface area (TPSA) values of piperine and DOX were 38.770 and 206.070 Å^2^, respectively, which explain the good bioavailability of piperine. Moreover, piperine exhibited moderate-to-high permeability towards the Caco-2 cell line; however, DOX showed low permeability. Furthermore, in the conducted AMES toxicity investigation, piperine tested negative for mutagenic adverse effects in comparison with DOX, which showed toxicity **(**Table 2 and Fig. 2).

**Table 2:** ADMET features of piperine and doxorubicin (DOX)

	Acceptable ranges	Piperine	DOX
Molecular weight(g/mol)	≤ 500	285.140	543.170
nHA	*≤* 12	4	12
nHD	*≤* 7	0	7
nRot	≤ 11	4	5
logP	< 5	2.841	2.012
Lipinski’s rule	Accepted	Accepted	Rejected
TPSA	≤ 140	38.770	206.070
Caco-2 permeability	>−5.15 Log unit	−4.839	−6.086
AMES Toxicity	Nontoxic	Nontoxic	Toxic

nHA: Number of hydrogen bond acceptors; nHD: Number of hydrogen bond donors; nRot: Number of rotatable bonds; Logp: octanol/water partition coefficient; and TPSA: Topological Polar Surface Area

### Cytotoxicity of piperine against breast cancer cells

**Fig. 3:**
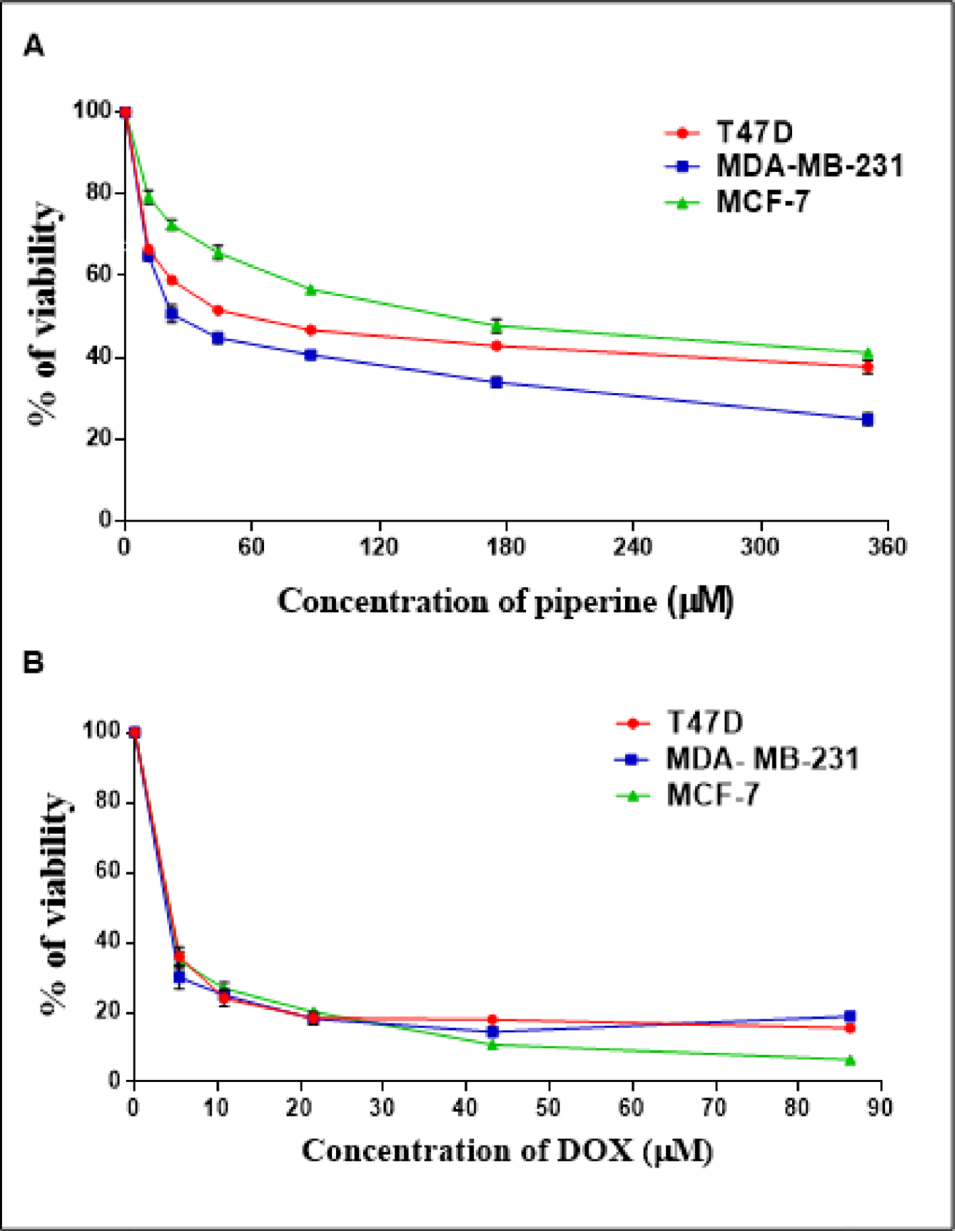
Different cancer cell lines are inhibited in their growth by piperine (**A**) and doxorubicin (DOX) (**B**). Cells were dosed for 48 h with varying concentrations of each drug, and cell viability was plotted versus drug concentration to determine the IC_50_. The IC_50_ values for each drug were given as the mean ± SE of three separate biological triplicate experiments

Our initial assessment focused on evaluating the antiproliferative effects of piperine and DOX using the commonly employed MTT assay against a range of breast cancer cell lines. Piperine exhibited a notable inhibition of cancer cell growth, with IC_50_ values of 158.1 ± 3.32, 32.37 ± 2.5, and 68.15 ± 3.52 µM against MCF-7, MDA-MB-231, and T47D, respectively. Notably, these findings underscored piperine’s suppressive influence on various types of breast cancer cells, with its most pronounced anticancer effect observed against MDA-MB-231 cells. This impact was compared to the reference chemotherapy, DOX, which exhibited an IC_50_ of 0.278 ± 0.96 µM, as portrayed in Fig. 3.

### Cell cycle investigations

**Fig. 4:**
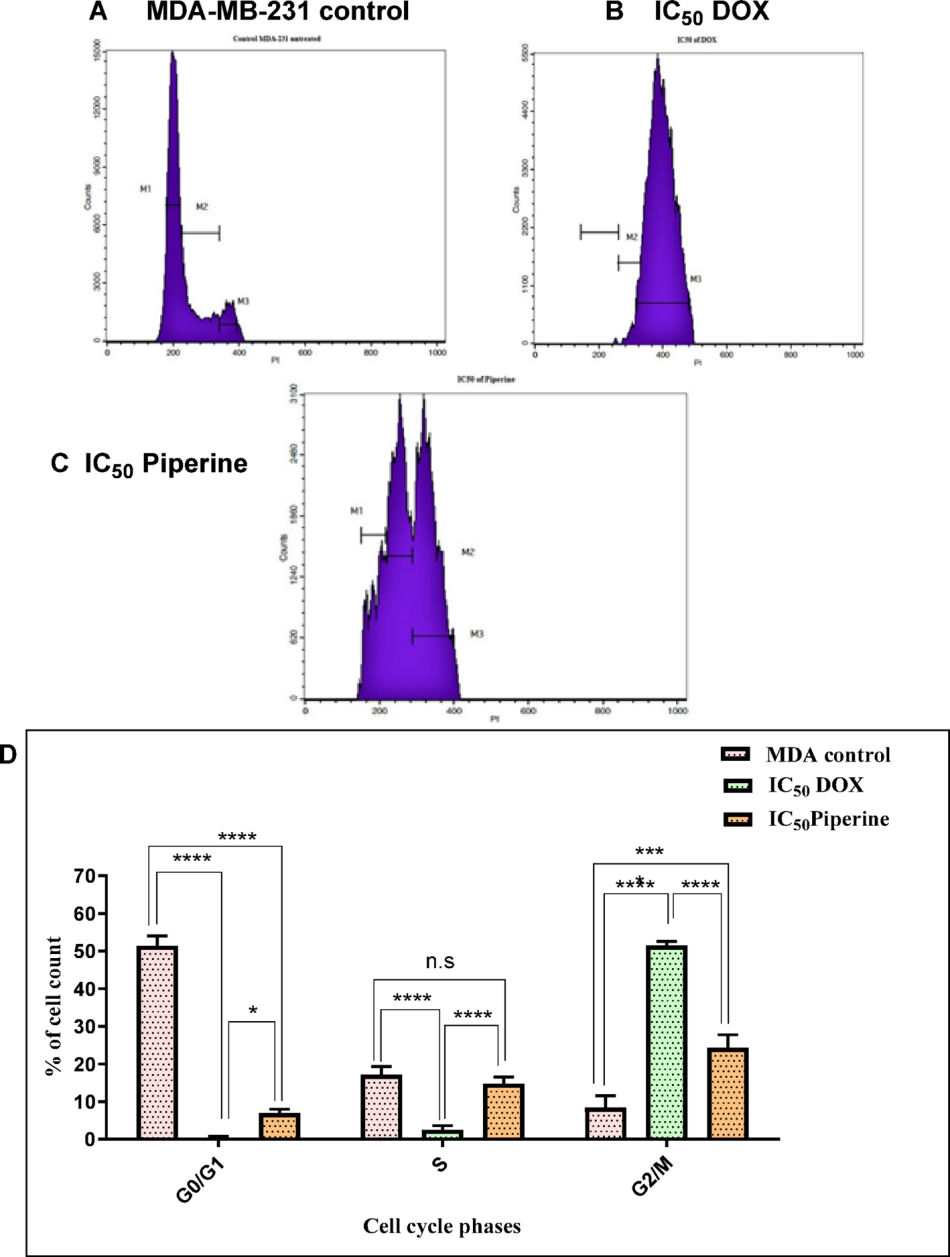
Flow cytometric analysis of MDA-MB-231 cell cycle phases. **A** control MDA-MB-231. **B **MDA-MB-231 treated with IC_50_ of DOX. **C** MDA-MB-231 received IC_50_ of piperine **D** Statistical analysis results, where results were expressed as mean ± SE, and (n.s) is non-significant and ^*^p < 0.05; ^****^p < 0.0001 values were significant vs. MDA-MB-231 control group

The impact of piperine and DOX on the distribution of MDA-MB-231 cells was scrutinized through the utilization of flow cytometry and PI staining. Intriguingly, treatment with the IC_50_ concentrations of piperine and DOX induced a significant alteration in cell distribution, characterized by a noticeable accumulation of cells (24.3 ± 3.4% and 51.5 ± 1%, respectively) in the G2/M phase in comparison with untreated cells (8.39 ± 3.2%), as vividly demonstrated in Fig. 4.

### Immunoblotting evaluations

**Fig. 5:**
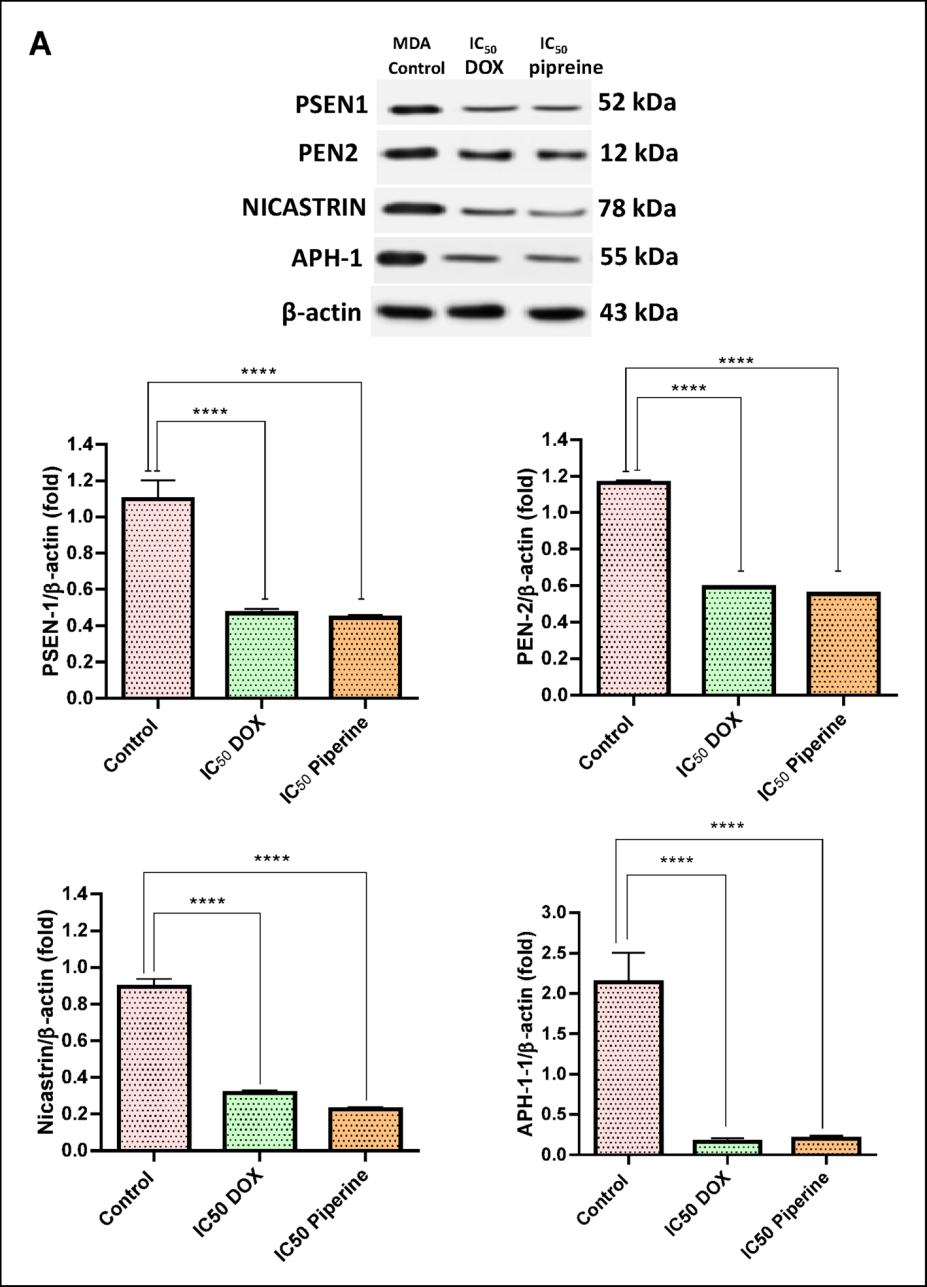

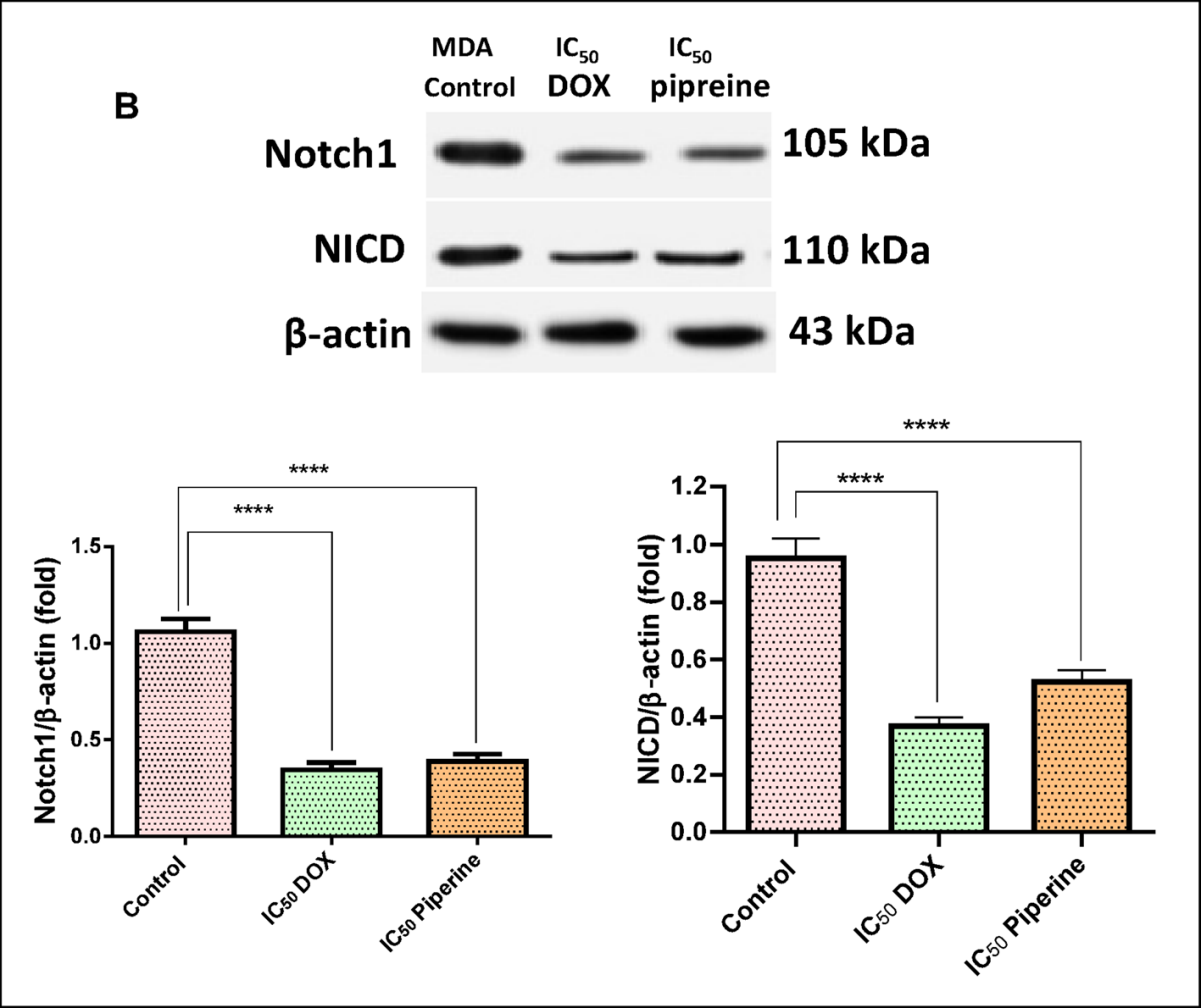

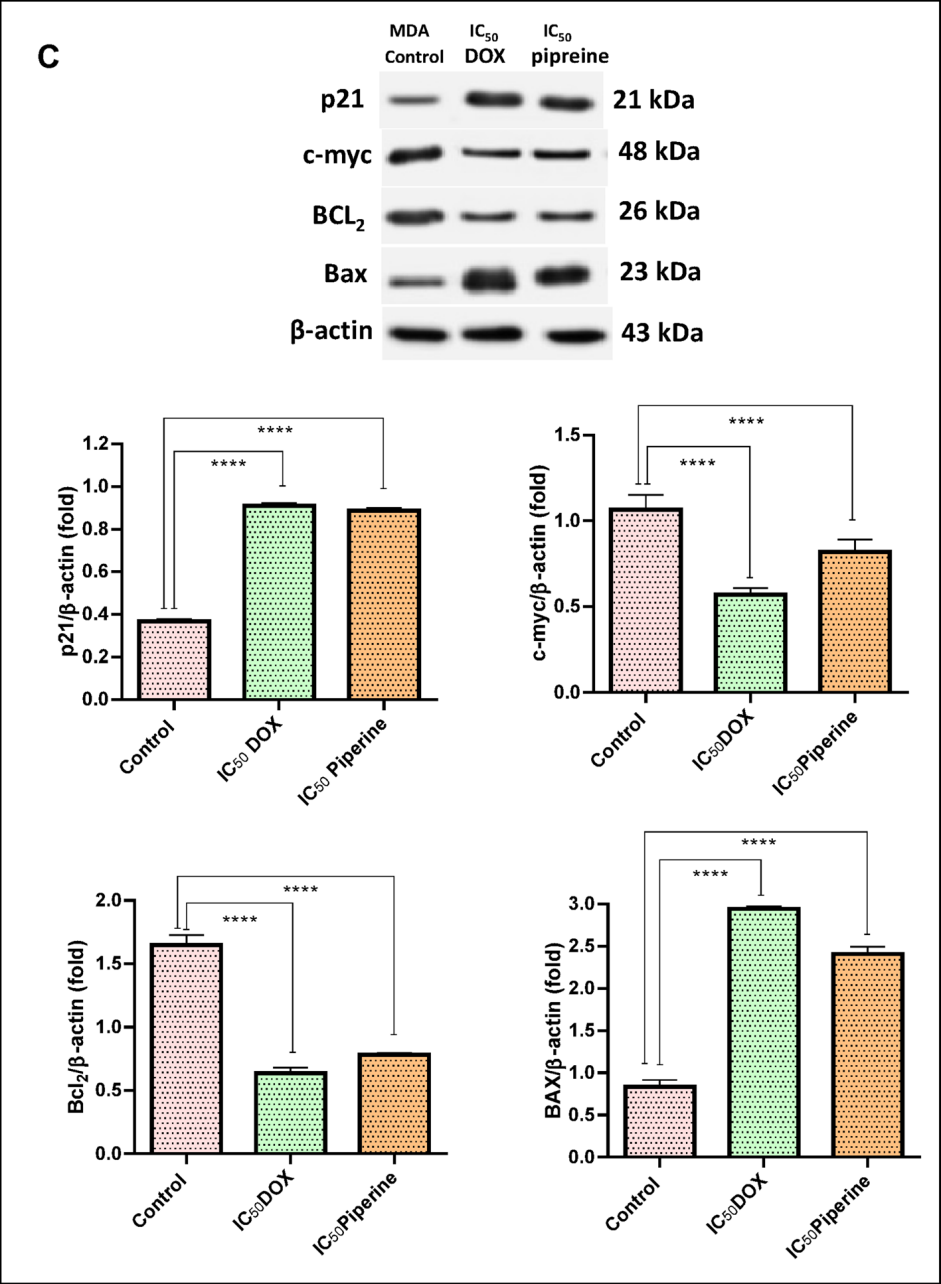

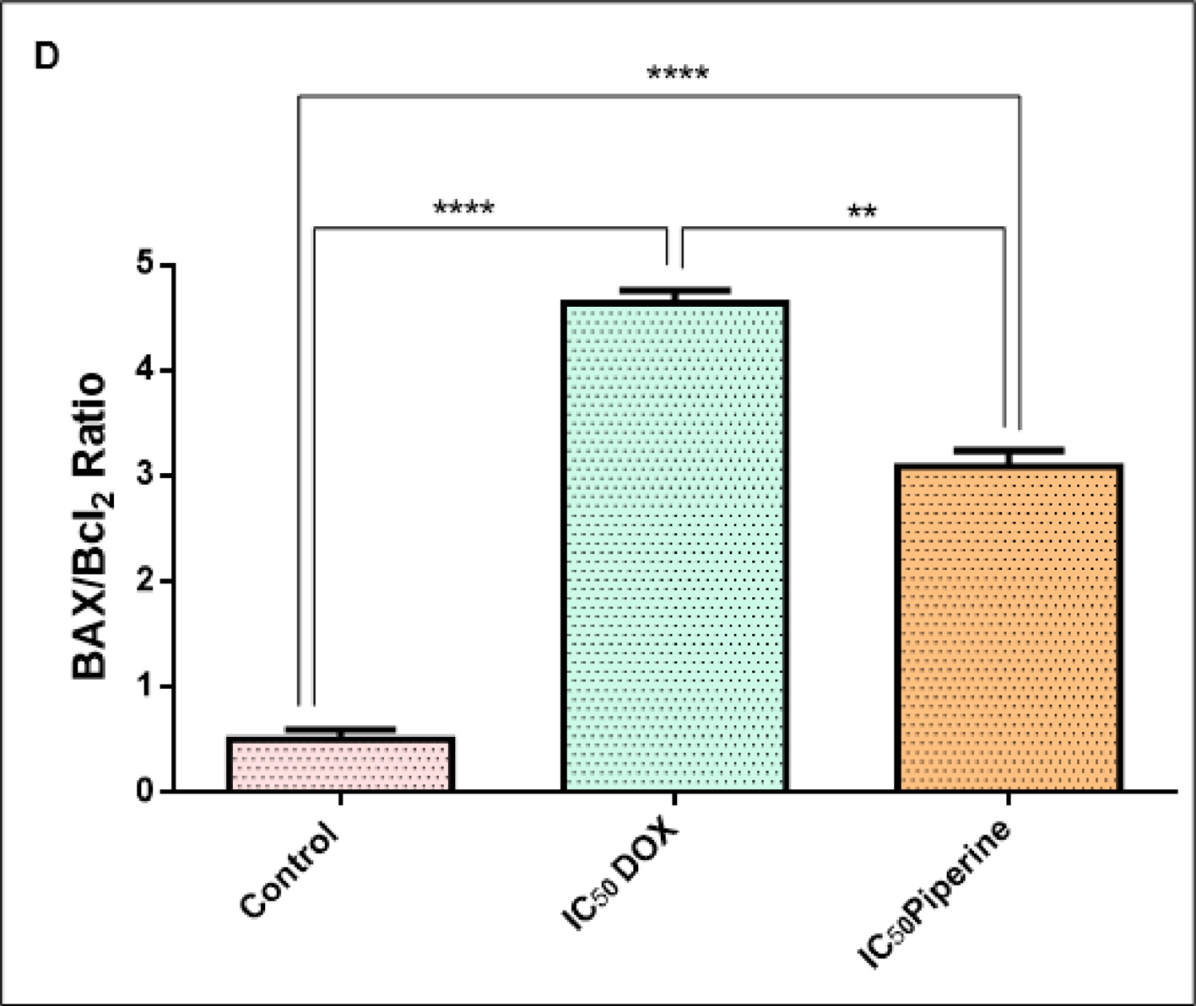
Immunoblotting analysis of γ-secretase/Notch axis in MDA-MB-231 breast cancer cells treated with IC_50_ of piperine or DOX for 48 h **A** The influence of piperine and DOX on γ-secretase subunits. **B** The inhibitory effect of piperine and DOX on Notch-1 receptor and NICD **C** The downregulation effect of piperine and DOX on Notch target proteins and apoptosis interrelated proteins (p21, c-myc, Bcl-2, and Bax). **D** Bax/Bcl-2 ratio. Results were expressed as mean ± SE, and ^**^p < 0.01; ^****^p < 0.0001 values were significant vs. the MDA-MB-231 control group

In this section, we probed the expression of proteins within the Notch pathway and its associated components, as depicted in Fig. 5 (A-C) and Figs. S1-S12. Our findings unveiled that treatment with the IC_50_ concentrations of DOX or piperine significantly inhibited the γ-secretase protein complex (PSEN-1, PEN-2, NICASTRIN, and APH-1) (*p* < 0.0001) compared to untreated cells. Consequently, this inhibition of γ-secretase activity led to the suppression of Notch1 and its cleavage process, thereby significantly reducing NICD levels (*p* < 0.0001) in comparison to untreated cells. The blockade of the Notch pathway further manifested in a conspicuous decline in the Notch target protein c-myc, coupled with a notable upregulation of p21 (*p* < 0.0001) when compared to control cells. Additionally, the treatment with piperine exhibited a potential rise in Bax expression, along with a reduction in Bcl-2 levels (*p* < 0.0001), which was confirmed to have pro-apoptotic effects through calculating the BAX/Bcl-2 ratio in comparison to untreated cells, as found in Fig. 5 (D).

## Discussion

The incidence and progression of breast carcinoma have a significant influence on females’ health [23]. Chemotherapy is the top choice for breast cancer suppression. However, the usage of chemotherapeutic drugs is severely constrained due to their non-selectivity, side effects, and resistance [24]. Hence, natural products gained increased attention as anticancer compounds attributable to their low toxicity and great efficacy [25]. The alkaloid compound piperine present in black and long pepper affects the growth and survival of different malignancies, particularly breast carcinoma [14]. Prior in vitro studies demonstrated that, in contrast to cancer cells, piperine had a less harmful impact on healthy human cells like osteoblasts, fibroblasts, and mammary cells [26–28].

Molecular docking is widely used in the search for targeted therapies, as it enables reliable and rapid predictions of protein-ligand complex binding energies and biomolecular conformations. In this study, piperine and DOX were docked into γ-secretase, which is essential in Notch signaling activation. Our simulations suggested that piperine bonded via hydrogen interaction; however, DOX bonded via hydrogen and non-covalent bonds. Both piperine and DOX interacted with ARG B115, which may serve as a key anchoring residue within the binding pocket. Additionally, electrostatic interactions with residues such as ASP A695, ILE A690, TYR B119, and ALA 694 imply that both compounds occupy a similar spatial region within the active site. The presence of overlapping residues suggests that both ligands target a common functional region of γ-secretase, potentially influencing its catalytic activity. Since γ-secretase mediates cleavage within the Notch signaling pathway, shared anchoring interactions may point toward a convergent modulatory mechanism. These theoretical predictions, when considered alongside our empirical observations, suggest that the inhibition of γ-secretase could be a potential mechanism contributing to the anticancer effects of piperine and DOX.

The online tool, ADMET, is used to assess a drug’s bioavailability and effectiveness, which is essential for elucidating clinical studies of therapies [29]. Lipinski’s “rule of five” guides the design of compounds that are intended to be administered orally. It sets limits on attributes including molecular weight, the number of hydrogen-bond donors and acceptors, and the partition coefficient (logP), beyond which poor oral activity is anticipated [30]. In this work, piperine follows Lipinski’s rule. Additionally, a crucial chemical characteristic in drug development, TPSA, helps predict a drug’s ability to cross biological membranes and is especially important for oral bioavailability. The TPSA must be lower than 140 Å^2^ to have a high oral bioavailability [31]. The results exhibited good oral bioavailability, whereas the TPSA value of piperine was 38.770 Å^2^. Moreover, piperine showed a good permeability towards Caco-2 cells. A drug candidate has to pass a toxicity risk assessment before being considered for medication development [32]. The fact that piperine tested negative in AMES toxicity means that it has no mutagenic properties. Hence, a promising anticancer treatment candidate, piperine, demonstrated excellent in-silico physicochemical and bioavailability without any toxicity.

In this study, the cytotoxicity of piperine and DOX on different human breast carcinoma cell lines (MCF-7, T47D, and MDA-MB-231) was studied via MTT assay. Piperine inhibited cancer cell growth with the greatest inhibitory impact against MDA-MB-231 cells, which agrees with previous studies [33, 34]. DOX also exhibited an effective anticancer impact towards MDA-MB-231 cells, which fits with a previous study [35]. Therefore, MDA-MB-231 cells were used to expand this work.

Prior investigations indicated that cancer cell cycle checkpoints are ineffectively controlled [36]. Therefore, cell cycle analysis was performed on MDA-MB-231 cells to learn more about how piperine and DOX suppress tumor growth. Here, each of the piperine and DOX stopped the cell cycle of MDA-MB-231 cells at the G2/M phase, which is consistent with previous studies [26, 37]. These findings are in line with a previous study, which reported that piperine upregulates the expression of cell cycle inhibitor p21, inhibiting cyclin-dependent kinase 1 (CDK1)-cyclin B activity, which is needed for progression through the G2/M phase, halting cell cycle in cancer cells [38]. Additionally, DOX intercalates between DNA base pairs and suppresses topoisomerase II, stopping DNA from relaxing and re-ligating during replication. Double-strand DNA breaks are the result of this, and their accumulation halts the cell cycle progression by activating the G₂/M checkpoint [39]. Moreover, reactive oxygen species (ROS) produced by DOX damage DNA, which intensifies G₂/M arrest signals [40]. This suggests that piperine-induced G2/M arrest may occur primarily through regulation of cell cycle–associated proteins rather than direct genotoxic stress, while DOX induces G2/M arrest via a DNA-damaging mechanism.

Notch signaling promotes cell survival and regulates cellular stress responses. Therefore, inhibiting the Notch pathway may help limit tumor proliferation [6]. Mammals have distinct notch receptors (1 to 4). Notch has been proven to be an oncogene in breast cells, so the overexpression of Notch1, 3, or 4 is sufficient to turn healthy breast epithelial cells into cancerous ones. For breast cancer patients, overexpression of Notch1 predicts worse overall survival outcomes [41]. In the present study, treatment with piperine or DOX downregulated Notch1 protein expression in MDA-MB-231 cells, demonstrating their inhibitory role on the Notch signaling. These findings agree with recent research demonstrating that piperine inhibited the Notch pathway by down-regulating Notch1 and Jagged1 signaling, besides decreasing the expression of downstream effectors Hey1 and the Hes family bHLH transcription factor 1 in docetaxel-resistant prostate cancer cell line (DU145/DTX) [42]. Additionally, the combination of curcumin, piperine, and sorafenib suppressed MCF-7 survival via downregulating Notch gene expression [43]. Although the effect of DOX on Notch signaling in malignant cells varies depending on the cancer type, drug dosage, and therapy duration, the majority of research suggests that it has an anti-proliferative, pro-apoptotic impact by modifying Notch activity. DOX was found to trigger apoptosis in breast neoplastic cells via decreasing Notch1 and Hes1 expression, particularly in triple-negative lines like MDA-MB-231 [44]. Moreover, the notch pathway is a tumor cell resistance regulator, and blocking its activity increases breast cancer cells’ sensitivity to anticancer medications [45–47].

In the Notch pathway, a crucial protein lyase that opens downstream gene transcription is γ- secretase [48]. Five fundamental proteins make up this multi-subunit protein complex, which is integrated into the cell membrane: anterior pharynx-defective (APH-1), presenilins (PSENs; PSEN-1 and PSEN-2), presenilin enhancer (PEN-2), and NICASTRIN (NCSTN) [49]. PSEN-1 and PSEN-2 serve as the catalytic core for building the γ-secretase complex [50]. NCSTN maintains PSENs’ stability, which regulates the complex’s intracellular transport and takes part in substrate recognition. After proteolysis, PEN-2 contributes to complex stability. However, APH-1 promotes its proteolytic activity [51]. When the ligand attaches to the Notch receptor, the γ-secretase cleaves it, releasing NICD, which triggers the translocation into the nucleus, promoting target gene transcription. As a result, blocking γ-secretase activity is reported to stop the Notch receptor cleavage and thereby block Notch signaling [52]. In this study, piperine repressed the Notch pathway by downregulating γ-secretase subunit expression in MDA-MB-231 cells. The inhibitory effect of piperine on γ-secretase activity could be explained by inhibiting the catalytic subunit of γ-secretase, PSEN-1, and thereby blocking the Notch protein cleavage mediated by PSEN-1. Here, these results are confirmed by piperine’s ability to downregulate NICD protein expression in MDA-MB-231 cells. DOX also downregulated γ-secretase subunits and NICD protein expression in MDA-MB-231 cells. A prior study reported that a γ-secretase inhibitor increases sensitivity to DOX in MDA-MB-231 cells [44].

There are many Notch target genes; some are tissue-specific, while others depend on Notch signaling in various tissues. In breast carcinoma, the Notch pathway is known to regulate the transcription of genes involved in cell cycle control, including cyclins like cyclin D1 and cell cycle regulators such as p21 and p27. Dysregulation of Notch signaling has been associated with uncontrolled proliferation and tumor progression [53]. A Previous study indicated that Notch1 downregulation inhibited cancer cell growth through the upregulation of p21 [54]. Moreover, inhibition of γ-secretase prevents Notch receptor cleavage and activation, thereby suppressing downstream transcriptional programs that promote cell cycle progression. Several studies have reported that attenuation of Notch signaling can lead to cell cycle arrest at G2/M through modulation of cyclin B1, CDK1, and checkpoint regulators [55, 56]. In this study, piperine upregulated p21 protein expression, thereby arresting the MDA-MB-231 cell cycle, which agrees with a previous study [38]. DOX was also found to upregulate p21, halting the cell cycle at G2/M phase in MDA-MB-231 cells, which is consistent with a previous study [57].

On the other hand, Notch1 was found to directly activate several biosynthetic pathways and induce *c-myc* gene expression, leading to malignant cell growth [58]. Here, piperine inhibited c-myc protein expression in MDA-MB-231 cells, which may be due to the piperine’s efficacy to inhibit Notch1 expression, and agrees with a previous study [59]. In the current study, DOX also suppressed c-myc protein expression in MDA-MB-231 cells. Suppression of c-myc enhances the cytotoxic impact of DOX in breast cancer cells [60].

Reduced expression of pro-apoptotic proteins and increased production of anti-apoptotic proteins are the roles of the Notch pathway [61]. Aberrant Notch activation promotes tumor cell survival through upregulation of anti-apoptotic proteins such as Bcl-2 and survivin and activation of pro-survival pathways such as PI3K/AKT. Conversely, inhibition of Notch, often via blockade of γ-secretase, has been shown to induce apoptosis and suppress tumor growth [55, 62]. Bcl-2 regulates mitochondrial permeability and is the gene that Notch uses to control mitochondrial function and cell apoptosis [63]. In this work, piperine significantly upregulated Bax and downregulated Bcl-2 protein expression, thereby triggering apoptosis via suppressing the Notch pathway in MDA-MB-231 cells. These results are consistent with previous research indicated that piperine upsets the Bax and Bcl-2 balance in breast neoplastic cells [64]. Piperine’s capacity to disrupt the structure of the mitochondrial membrane and consequently cause cytochrome c and Smac/DIABLO to leak out of the mitochondrial cytoplasm was further attributed to its apoptotic effect on breast cancer cells [13]. The current study also showed that DOX upregulated Bax and downregulated Bcl-2 protein expression, leading to apoptosis in MDA-MB-231 cells, which agrees with previous work [65]. DOX was also found to alter the ratio of Bax to Bcl-xL in breast cancer cells [66].

It is important to note that piperine exhibited a lower anticancer potency compared to DOX, requiring higher concentrations to achieve comparable cytotoxicity. This difference is anticipated, as DOX is a potent, standardized chemotherapeutic agent that acts directly as a genotoxic compound. Conversely, piperine is a plant-derived alkaloid that functions through pleiotropic mechanisms, including the modulation of signaling pathways like Notch. While piperine possesses lower cytotoxic potency, its therapeutic value lies in its high safety. Furthermore, the clinical significance of piperine may not be as a standalone replacement for DOX, but as a chemosensitizer that may lower the required dose of DOX, thereby reducing systemic toxicity and cardiotoxicity associated with high-dose chemotherapy.

## Conclusion

According to our investigation, the anticancer potential of piperine and DOX against breast cancer cells was manifested through cell cycle arrest and Notch pathway inhibition, specifically by suppressing Notch 1 and γ-secretase expression. This inhibition further modulates key downstream targets, including p21 and c-myc. Moreover, each of these drugs triggered apoptosis by upregulating the apoptotic Bax and downregulating the anti-apoptotic Bcl-2 protein expression in breast cancer cells. These findings suggest that piperine acts as a potent bioactive compound for breast carcinoma treatment.

## Limitation statement

A limitation of the current study is the absence of direct evaluation on normal cell lines. However, it is noteworthy that the piperine used in our experiments was of high analytical purity, ensuring the observed effects are substance-specific. Moreover, the safety profile of piperine is well-established in previous studies, which reported that piperine exhibits selective cytotoxicity, effectively targeting malignant cells while sparing normal cells. These established findings, combined with our mechanistic data, support the potential of piperine as a safe therapeutic candidate. Furthermore, in vivo and clinical studies are required to prove its therapeutic efficacy and safety profile.

## Supplementary Information

Supplementary Material 1.

## Data Availability

The datasets generated and/or analyzed during the current study are available in the following repositories: Protein structures: The macromolecular structures used for molecular docking are available in the Protein Data Bank (PDB) repository under accession numbers: [(PDB: #6IYC)](https:/www.rcsb.org/structure/6IYC) ([https://www.rcsb.org/structure/6IYC](https:/www.rcsb.org/structure/6IYC)). Computational prediction data: [ADMETlab 2.0] (https:/admetmesh.scbdd.com/service/screening/index) ([https://admetmesh.scbdd.com/](https:/admetmesh.scbdd.com)). Cell lines: All cell lines were obtained from the American Type Culture Collection (ATCC): MCF-7 [(#ATCC HTB-22)](https:/www.atcc.org/products/htb-22), T47D [(#ATCC HTB-133)](https:/www.atcc.org/products/htb-133), and MDA-MB-231 [(#ATCC HTB-26)](https:/www.atcc.org/products/htb-26). Other data: All remaining data supporting the findings are included in the article and its supplementary materials.
