# Supplementary material for: Antineoplastic effect of piperine compared with doxorubicin via suppressing γ-secretase/notch pathway in breast cancer cells
Source: Discov Oncol. 2026 Jul 12;17:1011. doi: 10.1007/s12672-026-05165-z (PMC13357466; doi:10.1007/s12672-026-05165-z)
Supplement: Supplementary file 1 — Supplementary Material 1. [file 12672_2026_5165_MOESM1_ESM.docx]

**Supplementary Materials**

**Piperine as a potent inhibitor of** **γ-secretase/Notch pathway in breast cancer cells**

Maha M. Salem^1^, [Hamed A. Abosharaf](https://sciprofiles.com/profile/1307351)^1^, [Marian N. Gerges](https://www.nature.com/articles/s41598-022-26571-7#auth-Marian_N_-Gerges)^1^*, Mohamed A Abd El-Moneim^2^, Tarek M. Mohamed^1*^, Aliaa M. Radwan^1^

^1^Biochemistry Division- Chemistry Department-Faculty of Science-Tanta University-Tanta 31257- Egypt

Emails: [maha_salem@science.tanta.edu.eg](mailto:maha_salem@science.tanta.edu.eg), [hamed_biochenistry@science.tanta.edu.eg](mailto:hamed_biochenistry@science.tanta.edu.eg), [marian.nabil@science.tanta.edu.eg](mailto:marian.nabil@science.tanta.edu.eg), [tarek.ali@science.tanta.edu.eg](mailto:tarek.ali@science.tanta.edu.eg) , [alyaa_radwan@science.tanta.edu.eg](mailto:alyaa_radwan@science.tanta.edu.eg)

^2^Biochemistry Department, Faculty of Dentistry, Sinai University, Al-Arish, North Sinai, Egypt.

Email: [mohamed.elsafty@su.edu.eg](mailto:mohamed.elsafty@su.edu.eg)


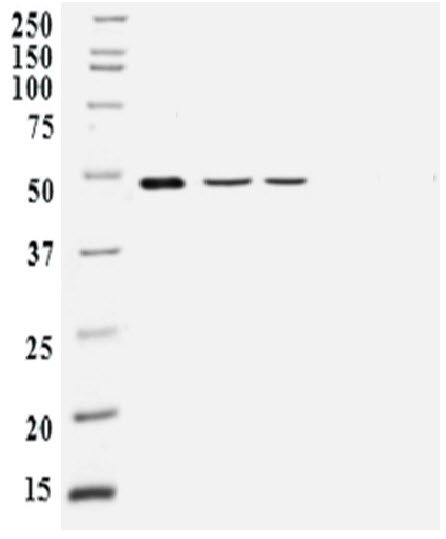


**M**

**Control IC_50_  IC_50_**

**DOX pipreine**

**Figure S1**: Raw data of PSEN-1 western blot gel before cropping.


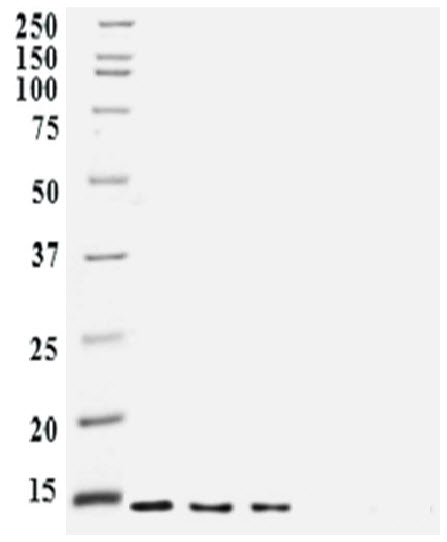


**M**

**Control IC_50_  IC_50_**

**DOX pipreine**

**Figure S2:** Raw data of PEN-2 western blot gel before cropping.


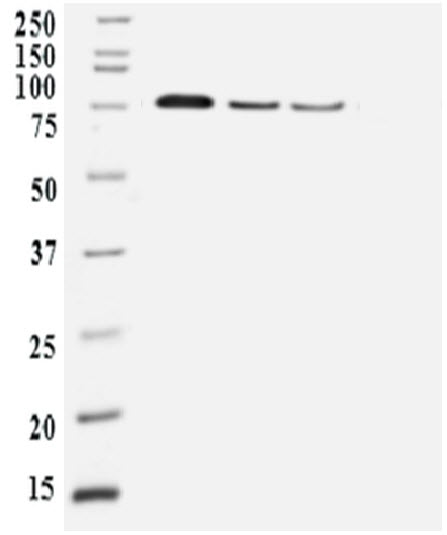


**Control IC_50_  IC_50_**

**DOX pipreine**

**M**

**Figure S3**: Raw data of NICASTRIN western blot gel before cropping.


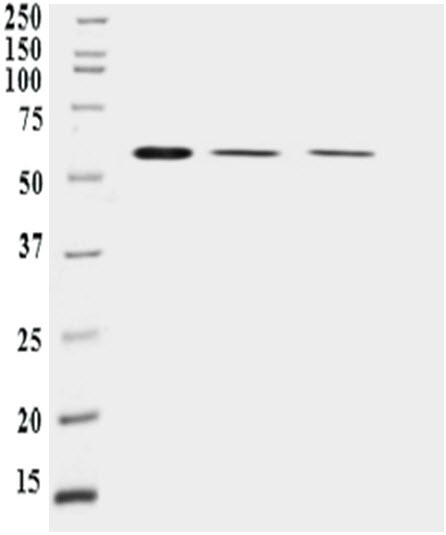


**Control IC_50_  IC_50_**

**DOX pipreine**

**M**

**Figure S4**: Raw data of APH-1 western blot gel before cropping.


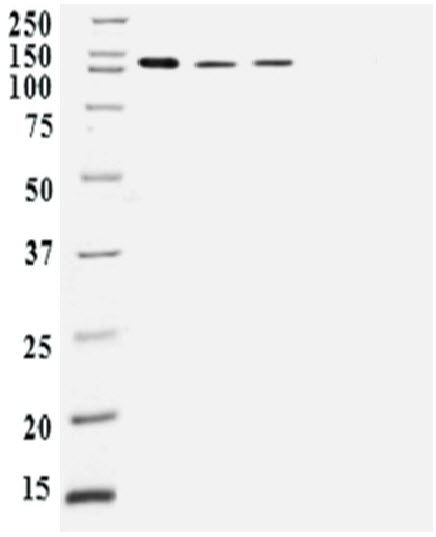


**Control IC_50_  IC_50_**

**DOX pipreine**

**M**

**Figure S5**: Raw data of Notch-1 western blot gel before cropping.

**M**

**Control IC_50_  IC_50_**

**DOX pipreine**


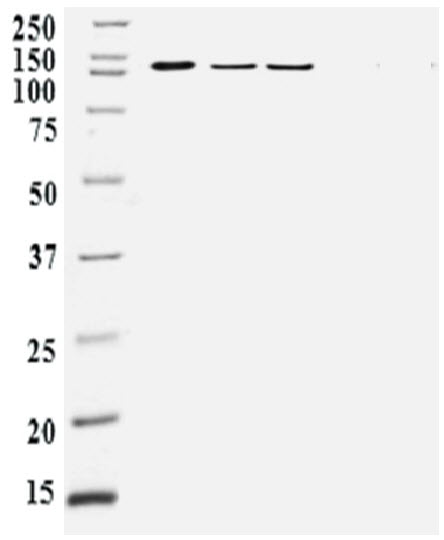


**Figure S6**: Raw data of NICD western blot gel before cropping.


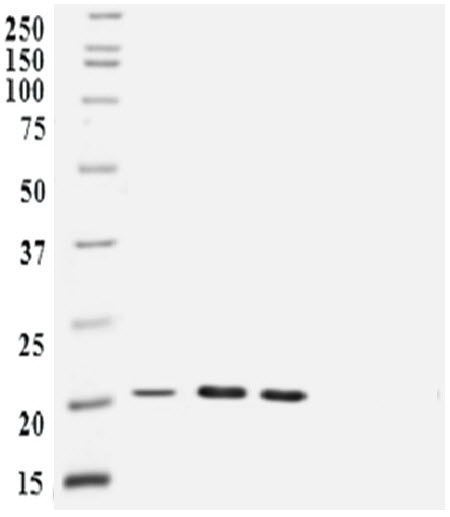


**Control IC_50_  IC_50_**

**DOX pipreine**

**M**

**Figure S7**: Raw data of p21 western blot gel before cropping.


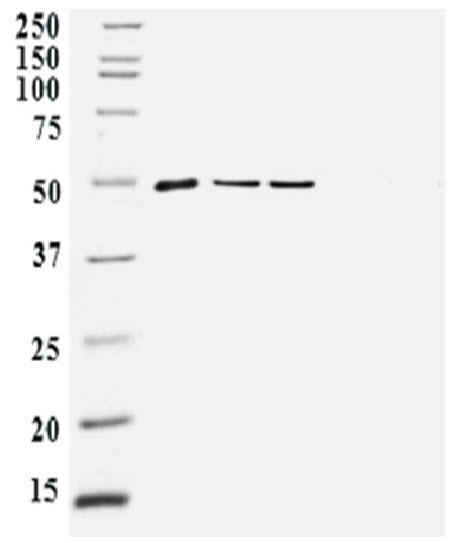


**Control IC_50_  IC_50_**

**DOX pipreine**

**M**

**Figure S8**: Raw data of c-myc western blot gel before cropping.


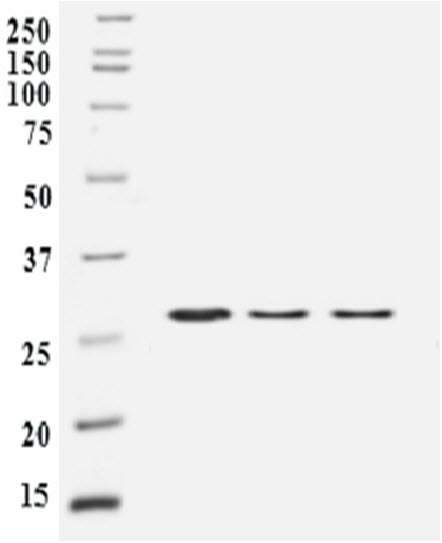


**Control IC_50_  IC_50_**

**DOX pipreine**

**M**

**Figure S9**: Raw data of Bcl-2 western blot gel before cropping.


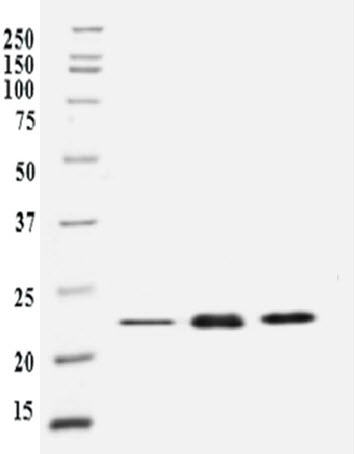


**Control IC_50_  IC_50_**

**DOX pipreine**

**M**

**Figure S10**: Raw data of Bax western blot gel before cropping.


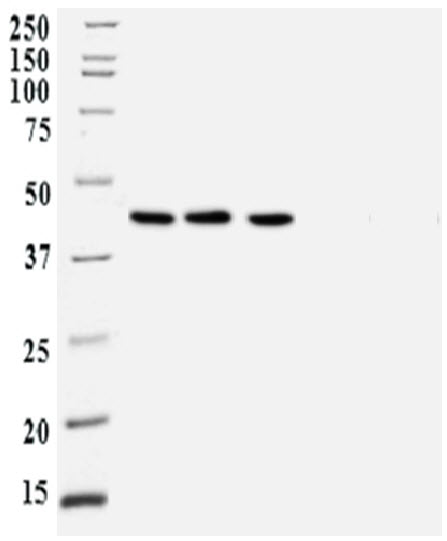


**Control IC_50_  IC_50_**

**DOX pipreine**

**M**

**Figure S11**: Raw data of β-actin western blot gel before cropping.


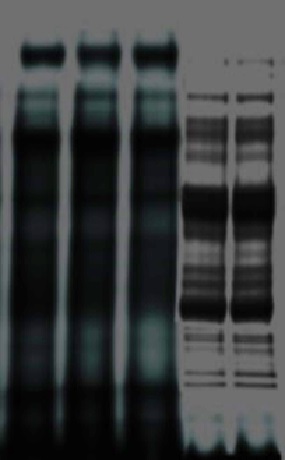


**Figure S12**: Raw data of western blot gel of all experimental samples.
